# Nighttime environmental noise and semen quality: A single fertility center cohort study

**DOI:** 10.1371/journal.pone.0240689

**Published:** 2020-11-04

**Authors:** Seung-Ah Choe, Seulgi Kim, Changmin Im, Sun-Young Kim, You Shin Kim, Tae Ki Yoon, Dae Keun Kim

**Affiliations:** 1 Department of Obstetrics and Gynecology, CHA Fertility Center Seoul Station, CHA University School of Medicine, Seoul, South Korea; 2 Department of Preventive Medicine, Korea University College of Medicine, Seoul, South Korea; 3 Graduate School of Public Health, Seoul National University, Seoul, South Korea; 4 Department of Geography, Korea University, Seoul, South Korea; 5 Department of Cancer Control and Population Health, Graduate School of Cancer Science and Policy, National Cancer Center, Gyunggi-do, South Korea; 6 Department of Urology, CHA Fertility Center Seoul Station, CHA University School of Medicine, Seoul, South Korea; Kaohsiung Medical University, TAIWAN

## Abstract

With increased population and urban development, there are growing concerns regarding health impacts of environmental noise. We assessed the relationship between nighttime environmental noise and semen quality of men who visited for fertility evaluation. This is a retrospective cohort study of 1,972 male patient who had undertaken semen analysis between 2016–2018 at a single fertility center of Seoul, South Korea. We used environmental noise data of National Noise Information System (NNIS), Korea. Using semiannual nighttime noise measurement closest to the time of semen sampling, individual noise exposures at each patient’s geocoded address were estimated with empirical Bayesian kriging method. We explored the association between environmental noise and semen quality indicators (volume, concentration, % of progressive motility, vitality, normal morphology, total motile sperm count, oligozoospermia, asthenozoospermia, and severe teratozoospermia) using multivariable regression and generalized additive models. Estimated exposure to nighttime environmental noise level in the study population was 58.3±2.2 L_eq_. Prevalence of oligozoospermia, asthenozoospermia, and severe teratozoospermia were 3.3%, 14.0%, and 10.1%. Highest quartile nighttime noise was associated with 3.5 times higher odds of oligozoospermia (95% CI: 1.18, 10.17) compared to lowest quartile. In men whose noise exposure is in 3^rd^ quartile, odds ratio (OR) of severe teratozoospermia was 0.57 (95% CI: 0.33, 0.98). The OR for 4^th^ quartile noise were toward null. In generalized additive model, the risk of oligozoospermia increases when the nighttime noise is 55 Leq dB or higher. Our study adds an evidence of potential impact of environmental noise on semen quality in men living in Seoul. Additional studies with more refined noise measurement will confirm the finding.

## Introduction

Environmental noise is defined as noise emitted from sources including road, rail and air traffic, industries, construction, and the neighborhood [1]. Exposure to environmental noise is so widespread that 30% of people are annoyed during the daytime and 20% complain disturbed sleep at night due to traffic noise [1]. By inducing acute cardiovascular and metabolic changes both directly and indirectly, exposure to environmental noise can lead secretion of stress hormones and blood pressure elevations caused by vasoconstriction [2]. Epidemiological studies showed environmental noise contributes to hearing loss, tinnitus, heart disease, stroke, anxiety, stress, depression, learning difficulties, job performance, sleep disorders, and reduced cognitive abilities [3, 4]. With urbanization and increasing population density, there are growing concerns regarding health impacts of environmental noise. In the western part of Europe, it is estimated at least one million healthy life years are lost every year from traffic-related noise [5].

Infertility is a disease of the reproductive system defined by the failure to achieve a clinical pregnancy after 12 months or more of regular unprotected sexual intercourse [6]. A male factor is responsible or contributory in 50–60% of infertility couples [7]. Idiopathic male infertility accounts for 50–75% of male infertility [8, 9]. The treatment options for these patients include lifestyle modification and empirical medical treatment, though the efficacy is limited. Identifying potential factors which is related with reduced semen quality would be important in early detection and prevention of male infertility. Because male reproductive function is highly sensitive to many physical agents generated by industrial activities [10, 11], a number of environmental components have been studied. Prior evidences indicated that exposures to ubiquitous substance such as endocrine disruptive chemicals and air pollutants are associated with reduced semen quality [12–14]. Recently, a study revealed that daytime and nighttime environmental noise is associated with higher risk of male infertility diagnosis without specifying abnormalities affected [15]. We aimed to assess association between environmental noise and semen quality parameters of men living in Seoul, the capital city of South Korea. This city is characterized by high population density, heavy traffic and intensive constructive activity, and thus shows substantial environmental noise compared to other areas nationwide. We analyzed the association between environmental noise and semen characteristics in men who visited a fertility center.

## Materials and methods

### Data

This study is a retrospective cohort study conducted among men who undertook semen tests between 2016–2018 at the CHA Fertility Center Seoul Station which is the largest single fertility center of the country located in Seoul, South Korea. Semen test was conducted as an initial evaluation in all couples who visited for a diagnostic purpose. The information of age, residential address, and underlying urologic diseases was obtained from medical records. Eligibility criteria include being married and aged 20 years or older. Excluding 189 patients diagnosed with varicocele, azoospermia, cryptorchidism, and known chromosomal abnormality and repeated test result (n = 378), we obtained initial semen analysis results of a total of 1,972 men who were living in Seoul at the time of examination (S1 Fig). We included only the first examination result of each patient. This study was approved by the institutional review board.

### Environmental noise

We used nighttime environmental noise data provided by the National Noise Information System (NNIS) of Korea (http://noiseinfo.or.kr). The NNIS is measuring environmental noise caused by transport, industrial, and recreational activities through noise monitors across the nation. They report manual measurements of environmental noise which are collected in 152 monitoring sites in Seoul. The measurement was conducted 2 times nighttime (10 PM to 6 AM) semiannually (from January to May and from July to November) for at least 5 minutes during a weekday (selected from Monday to Friday). Given that majority of patients spend their time outside of their home during daytime, we restricted our analysis to averaged value of nighttime noise to explore the association between environmental noise and sperm parameters. The level of noise was reported as the A-weighted equivalent continuous noise level, L_eq_ dB (A). It represents an average level of sound pressure within a certain time period.

We estimated environmental noise exposure at the geocoded residential address of each individual using spatial interpolation. Spatial interpolation methods such as inverse distance weighted (IDW), and kriging are commonly used in epidemiological studies to assess environmental exposure [16, 17]. Environmental noise in particular can be estimated using kriging which has been proposed as a suitable spatial interpolation method in producing noise maps covering the full temporal and spatial extent of the study area [18, 19]. In this study, we produced a noise map by spatial interpolation of noise data using empirical Bayesian kriging which presented the lowest root mean square error (RMSE) values during the study periods compared to IDW and ordinary kriging (S1 Table). This is one of the most commonly used method that optimizes covariance parameters incorporating uncertainties in kriging [20]. The accuracy of noise maps produced by spatial interpolation methods such as kriging depends substantially on the number of points measured as well as the spatial distribution of sites. The number of monitoring sites was 152 in 2016–2018 which is sufficient to cover subject locations across Seoul (S2 Fig). For geospatial analyses, we used ArcGIS Desktop v. 10.5 (ESRI, Redlands, CA).

### Semen collection and assessment

Patients were asked to produce semen samples in our Andrology laboratory by masturbation into a sterile plastic cup after 3 to 5 days of sexual abstinence. The semen specimen was left for 30 minutes at room temperature (22°C–24°C) for liquefaction. General semen quality parameters were assessed based on the 2010 World Health Organization (WHO) criteria [21]. In addition to measurement of semen volume, we assessed sperm concentration and motility Makler counting chamber, using ×200 polarized microscopy. The sperm morphology was analyzed after centrifugation of semen resuspended pellet which is dyed with Diff-Quik fixative solution, and immersion oil was dropped on the slide and evaluated using x1000 polarized microscopy. We assessed six continuous indicators (volume, sperm concentration, % of progressive motility, vitality, normal morphology, and total motile sperm count) obtained from semen analysis. Three dichotomous outcomes were defined as follows: oligozoospermia, sperm concentration as low as below 15 million/mL; asthenozoospermia, less than 32% of progressive motility; severe teratozoospermia, ≤1% normal spermatozoa with strict morphology criteria [22].

### Covariates

We included age and occupation at the time of semen analysis, season, fine particulate matter (PM_2.5_) and deprivation index in the model to control potential confounding effects [23–25]. We adopted the Korean Standard Classification of Occupations (KSCO) system in classifying the occupation of patients [26]. We re-categorized the variable by combining those categories having proportion of <2%. Season of semen sample collection was included in the analyses of sperm parameters to adjust for seasonality. We obtained district-average of PM_2.5_ which is calculated based on the measurements at maximum 40 regulatory air pollution monitoring sites in Seoul during 2016–2018 from the National Institute of Environmental Research as described in previous studies [27–29]. Three-month average concentrations of district-level PM_2.5_ before the date of semen test were used in the model because the positive association between air pollution and semen quality in previous studies was based on the exposures at least 90 days before semen sampling [30]. We adjusted for area-level of deprivation index to control the impact of neighborhood socioeconomic status. Deprivation index was calculated using 5-year census data including the proportion of car owners, population with education less than high school graduation, and single-person household [31]. More positive values of deprivation index mean higher degree of deprivation.

### Statistical analysis

We calculated median and quartile range or frequency (%) for demographic characteristics and semen quality parameters of study population. For environmental exposures, we presented mean and standard deviations. We normalized six semen quality parameters (volume, concentration, % of progressive motility, % of vitality, and % of normal morphology) using z-score based on the mean and standard deviation. Covariates included in the model were age and occupation at the time of semen analysis, season, PM_2.5_ and deprivation index. In exploring the association between environmental noise level and sperm parameters, we used quartiles of environmental noise measurement in multivariable linear regression model considering all covariates for six semen quality indicators. We calculated the odds ratios (OR) of three semen abrnomalities (oligozoospermia, asthenozoospermia, and severe teratozoospermia) per each quartile of noise exposure using the lowest quartile (Q1) as a reference. To examine the relationships between environmental noise and risk of a semen abnormality, we used generalized additive model with spline function of R (ver. 3.6.2).

## Result

Median ages of the study population and their partners were 38 and 37, respectively. Median duration of infertility was 1.3 years. Vast majority of men were white-collar and service workers (n = 1192, 65.1%). Average PM_2.5_ before semen test was 23.8±4.5 μg/m³ which is higher than the annual average (10 μg/m³) and lower than 24-hour mean target (25 μg/m³)of air quality guideline by WHO [32]. Mean district-level deprivation index of the study population was -0.5±3.0. Mean environmental noise level was 58.3±2.2 L_eq_ during nighttime which is higher than the national guideline for nighttime noise (40–55 L_eq_ for a residential area) [33]. Median concentration of sperm was 86 million/mL, and median proportions of progressive motility, vitality, normal strict morphology were 48%, 55%, and 3%, respectively. Prevalence of oligozoospermia asthenozoospermia, and severe teratozoospermia by strict morphology criteria were 3.3%, 14.0%, and 10.1%, respectively (Table 1).

**Table 1:** Characteristics of study population (N = 1,972).

Variables	Median	1^st^ quartile	3^rd^ quartile
Age of patient (years)	38	36	39
Age of partner (years)	37	34	39
Duration of infertility (years)	1.3	0.7	3
*Job category*^a^	**Frequency**	**Proportion**	
Managers, professionals and related workers	530	29.0%	
White-collar and service workers	1192	65.1%	
Sales, craft, equipment, machine operating workers and others	108	5.9%	
*Area-level covariates*	**Mean**	**SD**	
PM_2.5_ (μg/m³), monthly average	23.8	4.5	
Deprivation index	-0.5	3.0	
*Environmental noise at residential address*			
Nighttime (L_eq_ dB (A))	58.3	2.2	
*Semen quality parameters*	**Median**	**1**^**st**^ **quartile**	**3**^**rd**^ **quartile**
Concentration (million/mL)	89	58	127
Volume (mL)	2.8	2	3.9
Progressive motility (%)	48	38	56
Vitality (%)	55	48	62
Normal morphology (%)	3	1	4
Total motile sperm count (million)	116	66	180
Sperm abnormality	**Frequency**	**Proportion**	
Oligozoospermia (<15 million/mL)	65	3.3%	
Asthenozoospermia (<32% of progressive motility)	276	14.0%	
Severe teratozoospermia (<1%)	199	10.1%	

^a^We applied the Korean Standard Classification of Occupations to group the occupation of patients. The information of occupation was unavailable in 152 men.

When we grouped the population by quartiles of nighttime noise exposure, there were generally inverse association with higher quartiles of noise for volume, concentration, % of progressive motility, and vitality which did not reach statistical significance. Proportion of normal morphology was higher when the nighttime noise exposure was 3^rd^ and 4^th^ high quartile (Table 2).

**Table 2:** Adjusted regression coefficient of environmental noise (Leq dB (A)) for six continuous semen quality parameters^a^.

Parameters	Volume		Concentration		Progressive motility		Vitality		Strict Morphology		Total motile sperm count	
Quartiles of noise	Coefficient	P	Coefficient	P	Coefficient	P	Coefficient	P	Coefficient	P	Coefficient	P
Q1	0 (reference)		0 (reference)		0 (reference)		0 (reference)		0 (reference)		0 (reference)	
Q2	-0.01	0.934	-0.09	0.215	-0.02	0.745	-0.07	0.312	0.13	0.062	-0.09	0.195
Q3	0.04	0.629	-0.01	0.914	-0.06	0.452	-0.14	0.066	0.26	0.001	0.02	0.843
Q4	-0.01	0.898	-0.01	0.872	0.02	0.788	-0.03	0.719	0.22	0.013	0.04	0.660

^a^All six sperm parameters were standardized using z-score. Coefficient for each quartile shows change of the parameter from the z-score at lowest quartile of noise. Covariates included in the model were age and occupation at the time of semen analysis, season, fine particulate matter (PM_2.5_) and deprivation index.

**Fig 1:**
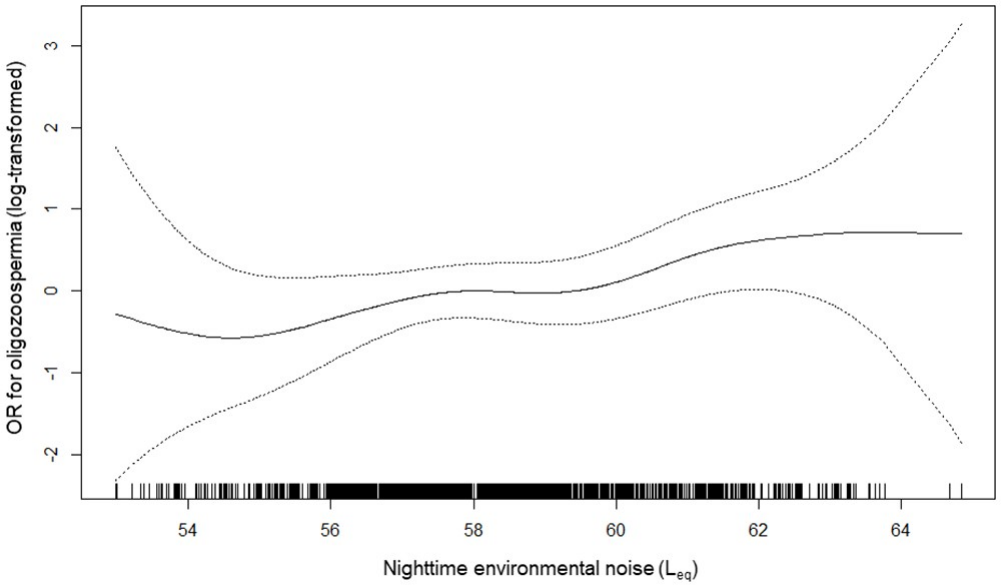
Relationship between nighttime environmental noise and odds ratio of oligozoospermia (log-transformed). Solid and dotted lines indicate smoothing spline lines and their 95% confidence intervals.

In the multivariable models for three semen abnormalities, highest quartile nighttime noise was associated with 3.5 times odds of oligozoospermia (95% CI: 1.18, 10.17) compared to lowest quartile (Table 3). In men whose noise exposure is in 3^rd^ quartile, OR of severe teratozoospermia was 0.57 (95% CI: 0.33, 0.98). The OR were toward null for 4^th^ quartile noise. Using generalized additive model, the risk of oligozoospermia increases when the nighttime noise is 55 L_eq_ dB or higher (Fig 1).

**Table 3:** Odds ratios for three semen abnormality per increase of nighttime environmental noise (n = 1,972).

Quartiles of noise	Oligozoospermia		Asthenozoospermia		Severe teratozoospermia	
	(<15 million/mL)		(<32% of progressive motility)		(<1% of normal morphology)	
	Unadjusted	Adjusted	Unadjusted	Adjusted	Unadjusted	Adjusted
Q1	1.00 (reference)	1.00 (reference)	1.00 (reference)	1.00 (reference)	1.00 (reference)	1.00 (reference)
Q2	1.83 (0.82, 4.11)	1.98 (0.84, 4.71)	0.82 (0.55, 1.22)	0.79 (0.52, 1.20)	0.67 (0.43, 1.06)	0.63 (0.39, 1.02)
Q3	1.00 (0.37, 2.70)	1.26 (0.44, 3.59)	1.08 (0.70, 1.65)	1.04 (0.66, 1.64)	0.56 (0.33, 0.92)	0.57 (0.33, 0.98)
Q4	2.95 (1.10, 7.91)	3.46 (1.18, 10.17)	0.76 (0.46, 1.26)	0.76 (0.44, 1.31)	0.78 (0.44, 1.38)	0.85 (0.47, 1.55)

Confidence intervals of each odds ratio in parentheses. Covariates included were age and occupation at the time of semen analysis, season, fine particulate matter (PM_2.5_) and deprivation index.

## Discussion

Oligozoospermia was more common where the nighttime environmental noise is 4^th^ quartile in men living in Seoul, Korea. There was positive association between nighttime noise and % of normal morphology. Association with other sperm parameters, asthenozoospermia, or severe teratozoospermia was not evident. Using a cohort of urban men who has a history of infertility, we observed potential impact of environmental noise at nighttime on male fertility.

The reduced sperm concentration in higher noise exposure has been postulated due to change in reproductive hormones which are involved in spermatogenesis. In an animal study, high environmental noise was associated with reduced sperm concentration and motility [34]. They also demonstrated that sperm count and progressive motility remained decreased after the noise exposure was discontinued supporting the findings of inhibited spermatogenesis in response to various stressors [22, 35]. Psychological stress has been known to affect spermatogenesis through altering the secretion of testosterone [36]. In rat, noise stress decreased testosterone levels while it increased the adrenocorticotropic hormone (ACTH) and cortisol levels [37]. In a study on men with unexplained infertility, low level of total testosterone was associated with reduced proportion of normal morphology, but was not associated with semen volume, sperm concentration and motility [38]. The mechanism of negative association of environmental noise with reduced sperm concentration warrants further study.

Prior studies revealed possible impact of environmental or occupational noise on semen quality mostly in occupational setting or general population. In an observational study, higher level of noise exposure was associated with lower concentration of testosterone, luteinizing hormone (LH), and follicle-stimulating hormone (FSH) in 27 healthy Iranian workers [39]. They observed also reduced sperm concentration, % of progressive motility, and normal morphology although the difference did not reach statistical significance probably due to small sample size [39]. Although self-reported occupational exposure to noise was not associated with semen quality [40], exposure to noise during work was observed to be associated with decreased motility and increased DNA damage of sperm [41]. A study of Min et al. reported higher OR for male infertility diagnosis when the estimated environmental noise exposure is higher for both daytime and nighttime using nationwide data [15]. We observed a counterintuitive association for % of normal morphology. It may be because our study population is restricted to hospital setting where the majority show normal morphology < 4%.

As a clinically based study, this study would require cautions in interpretation. There are possible residual confounding effects by unmeasured variables such as body mass index and health-related behaviors (smoking, alcohol drinking, strenuous exercise, etc.). Lack of information regarding these factors which may affect the semen quality can be another limitation of this study. However, considering these factors are potential mediators in the association between environmental noise and semen quality, adjusting for these factors may bias the effect estimates [42]. Second, as in other environmental health study, the estimation of noise exposure based on residential address and outdoor measurement might have been biased. Although our exposure estimation is based on noise measurement outside, prior studies showed good correlation between indoor and outdoor noise [43]. For the possible relocation of the patients during the exposure period, we believe future studies considering the information would be needed. If the extent of exposure misclassification occurs independent of health status and thus is non-differential, effect estimates are likely to be underestimated [44]. Furthermore, as we used semiannual data, some of the measurements might have been done after the sperm tests. Given that the monthly variation of environmental noise measured in 8 continuous monitors of Seoul is minimal [45], we believe the semiannual averages would provide valid estimates of chronic environmental noise exposure. When exploring the impact of highly variable exposures such as noise, perceived noise can be more valid exposure estimates [46], we suggest future studies would benefit from using additional information of perceived noise. We believe the consistent finding of this study with prior reports supports the method used in this study. Third, we do not have the information about the time they spent outdoors which would be another confounder in the association between noise and semen quality [7]. We believe our finding can be meaningful since we restricted our analysis to nighttime noise to which most of the patients had been exposed at their home [47]. Fourth, we could not specify the critical period of susceptibility because our estimation of noise exposure is based on semiannual measurement. Given the impact of environmental substance on the spermatogenesis can last for 4–12 months, our finding can still be valid in terms of chronic or sub-chronic exposure [48]. Lastly, our study population was from couples who visited an infertility clinic which may not be representative of general population. Considering that only limited number of men would undertake semen analysis in general, this study may have a value as a research investigating the association between environmental noise and sperm parameters.

## Conclusions

Our study adds an evidence of potentially detrimental impact of environmental noise on semen quality in men. This finding will be meaningful to clinicians and patients who are seeking for potential cause and preventive strategy of male infertility. Additional studies on this topic using more refined noise exposure measurement will confirm the finding.

## Supporting information

S1 Fig (TIF)

S2 Fig (TIF)

S1 Table Root mean square error (RMSE) values of three spatial interpolation methods by cross validation. Compared to IDW and ordinary kriging, empirical Bayesian kriging presents the lowest RMSE values during all study periods.
(DOCX)
