# Supplementary figures and images for: Nighttime environmental noise and semen quality: A single fertility center cohort study
Source: PLoS One. 2020 Nov 4;15(11):e0240689. doi: 10.1371/journal.pone.0240689 (PMC7641366; doi:10.1371/journal.pone.0240689)

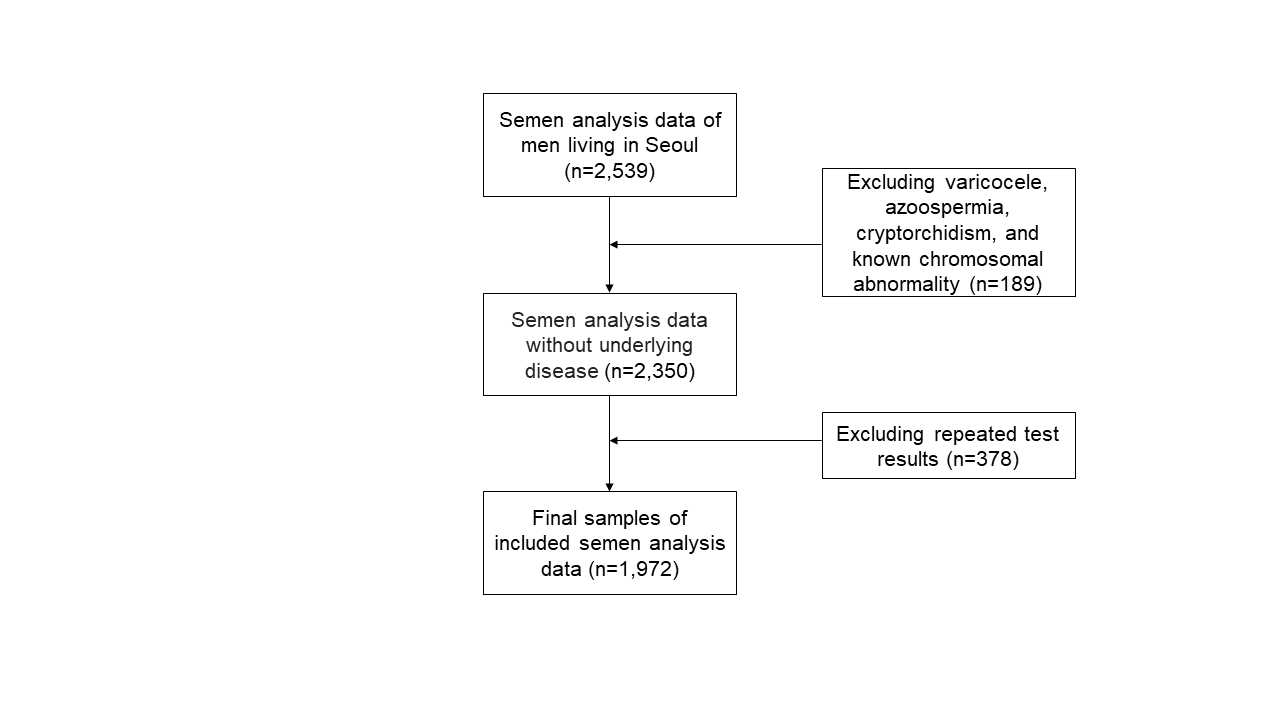

Supplement: S1 Fig — (TIF) [file pone.0240689.s001.tif]

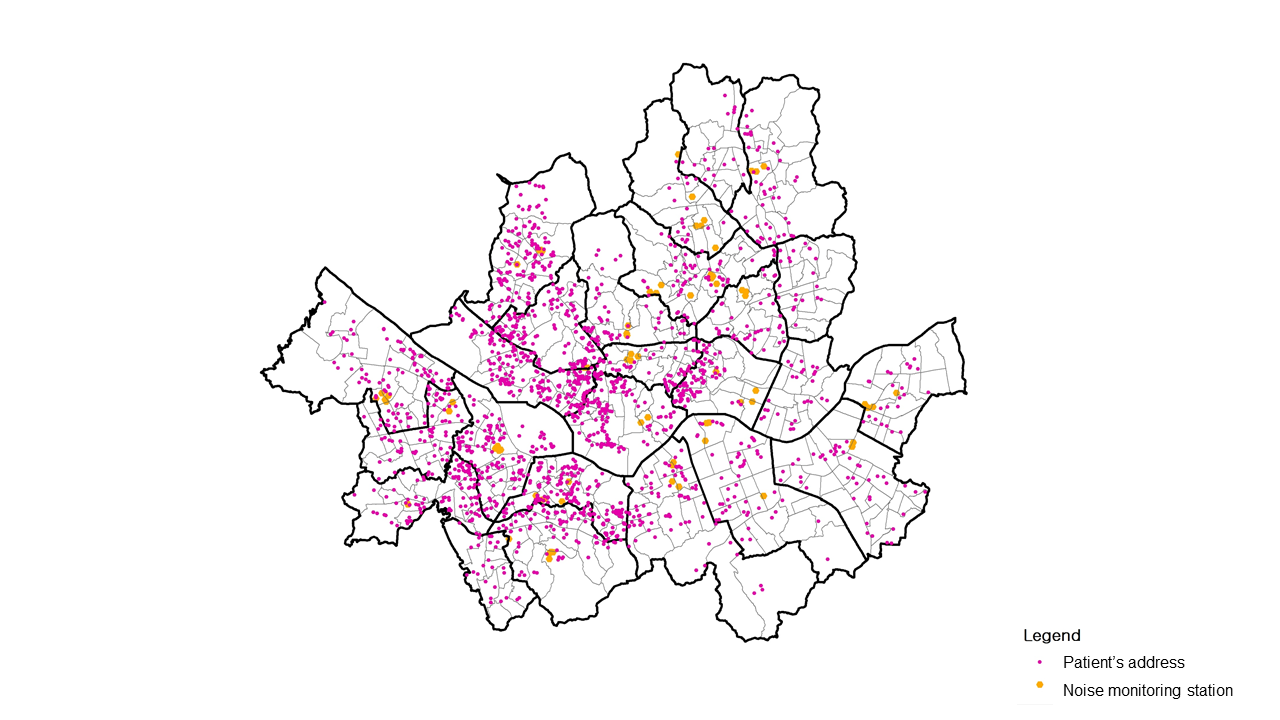

Supplement: S2 Fig — (TIF) [file pone.0240689.s002.tif]
