# Supplementary material for: Nighttime environmental noise and semen quality: A single fertility center cohort study
Source: PLoS One. 2020 Nov 4;15(11):e0240689. doi: 10.1371/journal.pone.0240689 (PMC7641366; doi:10.1371/journal.pone.0240689)
Supplement: S1 Table — Compared to IDW and ordinary kriging, empirical Bayesian kriging presents the lowest RMSE values during all study periods. (DOCX) [file pone.0240689.s003.docx]

S1 Table. Root mean square error (RMSE) values of three spatial interpolation methods by cross validation. Compared to IDW and ordinary kriging, empirical Bayesian kriging presents the lowest RMSE values during all study periods.

| Time | July to November, 2016 | January to May, 2017 | July to November, 2017 | January to May, 2018 | July to November, 2018 |
| --- | --- | --- | --- | --- | --- |
| Inverse Distance Weighting (IDW) | 9.62 | 10.32 | 10.17 | 10.02 | 9.65 |
| Ordinary Kriging | 8.96 | 9.49 | 9.40 | 9.37 | 8.99 |
| Empirical Bayesian Kriging | 8.87 | 9.29 | 9.18 | 9.16 | 8.82 |
